# Characteristics of Familial Lung Cancer in Yunnan-Guizhou Plateau of China

**DOI:** 10.3389/fonc.2018.00637

**Published:** 2018-12-18

**Authors:** Xiaojie Ding, Ying Chen, Jiapeng Yang, Guangjian Li, Huatao Niu, Rui He, Jie Zhao, Huanqi Ning

**Affiliations:** ^1^Key Laboratory of Lung Cancer Research of Kunming Medical University, Kunming, China; ^2^Yunnan Cancer Hospital and The Third Affiliated Hospital of Kunming Medical University & Yunnan Cancer Center, Kunming, China

**Keywords:** familial lung cancer, cancer susceptibility, Yunnan-Guizhou plateau of China, clinicopathologic characteristics, family history

## Abstract

**Background:** Lung cancer has inherited susceptibility and show familial aggregation, the characteristics of familial lung cancer exhibit population heterogeneity. Despite previous studies, familial lung cancer in China's Yunnan-Guizhou plateau remains understudied.

**Methods:** Between 2015 and 2017, 1,023 lung cancer patients (residents of Yunnan-Guizhou plateau) were enrolled with no limitation on other parameters, 152 subjects had familial lung cancer. Clinicopathologic parameters were analyzed and compared, 4,754 lung cancer patients from NCI-GDC were used to represent a general population.

**Results:** Familial lung cancer (FLC) subjects showed unique characters: early-onset; increased rate of female, adenocarcinoma, stage IV and other cancer history; unbalance in anatomic sites; all ruling out significant difference in smoking status. Unbalanced distribution of co-existing diseases or symptoms was also discovered. FLC patients were more likely to develop benign lesions (polyps, nodules, cysts) early in life, especially early-growth of multiple pulmonary nodules at higher frequency. Typical diseases with family history like diabetes and hypertension were also increased in FLC population. Compared to GDC data, our subject population was younger: the age peak of our FLC group was in 50–59; our sporadic group had an age peak around 60; while GDC patients' age peak was in 60–69. Importantly, the biggest difference happened in age 40–49: our FLC group and sporadic group had 3 times and 2 times higher ratio than GDC population, respectively. Moreover, the age peaks of our FLC males and FLC females were both in 50–59; while our sporadic females had the age peak in 50–59, much earlier than sporadic males (around 60–69); reflecting gender-specific or age-specific characters in our subject population.

**Conclusions:** Familial lung cancer in China's Yunnan-Guizhou plateau showed unique clinicopathologic characters, differences were found in gender, age, histologic type, TNM stage and co-existing diseases or symptoms. Identification of hereditary factors which lead to increased lung cancer risk will be a challenge of both scientific and clinical significance.

## Introduction

Lung cancer is often cited as a malignancy largely determined by environmental factors (1). However, Epidemiological surveys suggest that lung cancer show familial aggregation after proper adjustments for tobacco smoking and other environmental factors, indicating inherited genetic susceptibility is a typical feature of familial lung cancer (1–4).

Familial lung cancer (FLC) exhibit special features when compared with the sporadic counterpart, previous findings show similarity and diversity in different FLC populations, depending on the subjects' source. Even majority of them find an increased lung cancer risk in FLC population, the fold change vary from 1.3 ~3.5 (2–7). In addition, some support certain ethnic groups are affected more by inherited lung cancer susceptibility (6, 7); others find first-degree female relatives have a higher risk than first-degree male relatives (4, 8); and another suggests there seems to be a particular link between FLC and EGFR mutation in tumors of affected family members (3). In summary, genetic predisposition to lung cancer may be inherited with complex patterns in populations, and there can be unique characteristics within each population or subpopulation.

Lung cancer is a disease induced by interaction between genetic and environmental factors, and hypoxia is a typical feature of tumor microenvironment. Previous findings (9–11) suggest genetic adaptations to life at high altitude could potentially have different effects on human diseases in which hypoxia is a feature. Located in southwest China, Yunnan-Guizhou plateau has an average elevation of 2 kilometers (range: 1.5 ~ 4 kilometers). Populations living in high altitude regions (terrestrial elevation >2 kilometers) exhibit unique circulatory, respiratory, hematological and immune adaptations (12–15); beside phenotypes, the highlanders also vary in their genetic background (9, 10, 12, 16). Many features of high land residents have been characterized physiologically and pathologically (17, 18), especially cardiovascular disease and respiratory disease, in which lower oxygen of high elevation is considered as one key factor.

So it would be reasonable to predict: lung cancer, especially familial lung cancer of highland residents may harbor unique characters, when compared with general population. Current study is to investigate and characterize the clinicopathologic features of familial and sporadic lung cancer in China's Yunnan-Guizhou plateau, moreover provide insight into complex genetic susceptibility of lung cancer.

## Materials and Methods

### Patients

This trial was designed as a single-center real-world observational study. The lung cancer patients who enrolled in Department of Thoracic Surgery I of Yunnan Cancer Hospital from Jan. 2015 to Jan. 2017 were recruited. In order to investigate the characters of highland population with and without familial lung cancer, patients met the following criteria were selected: (1) The subject can be permanent native of Yunnan-Guizhou plateau; (2) If the subject's ancestor migrated from other provinces, the family has lived on this plateau for at least 3 generations. In total, 1023 lung cancer patients were enrolled with no limitation on other parameters. (3) Subject with familial lung cancer is defined as individual has three or more first-degree relatives affected by lung cancer. There were 152 patients who were classified as having familial lung cancer. All the information were based on self-report and confirmed by personal medical records.

Clinicopathologic data were documented in hospital cooperated databank (https://www.linkdoc.com), including age, gender, blood type, histologic type, family history etc. The TNM stage was reviewed according to the 8th edition of The International Association for the Study of Lung Cancer (IASLC) staging system. The majority of patients enrolled had adenocarcinoma (AD), squamous cell carcinoma (SCC) and small cell lung cancer (SCLC). Other co-existing diseases or symptoms were also documented, including: diabetes, hypertension, gallstone, gallbladder polyp, hepatic cysts, hepatic hemangioma, fatty liver, thyroid nodule, thyroid cysts, ovarian cysts, uterine myoma, renal cysts, renal stone and multiple pulmonary nodules. The study was approved by the Ethical Committees of Yunnan Cancer Hospital. All patients provided informed consent.

### Compare With Other Population

We first compared familial and sporadic lung cancer patients in our region; since non-plateau area data were not available, we chose GDC lung cancer data to represent a general population for further comparison. National Cancer Institute Genomic Data Commons (NCI-GDC) provide representative data on human cancers, including subjects of different ethnic groups. Since data on patients' living altitude was not available, subjects could come from all possible elevations. Basic information like gender, age and lung cancer histologic type were obtained from NCI-GDC (https://portal.gdc.cancer.gov). In total, 4754 lung cancer patients (male: 2401, female: 2349, unreported: 4) were included (Table S2). The data were compared with present work.

### Statistical Analysis

Chi-square test and Fischer's exact test were used to analyze the association of clinic-pathological parameters with familial lung cancer. SPSS 17.0 was used (SPSS Institute, Chicago, IL, USA). Statistical significance was set at *p* < 0.05 (two-sided p-value).

## Results

### Clinic-Pathological Features of Familial and Sporadic Lung Cancer in China's Yunnan-Guizhou Plateau

In total 1023 subjects were enrolled in the study, 152 were identified as familial lung cancer patients. The characters were listed in Table 1 and Table S1. The familial lung cancer (FLC) group included 94 (61.8%) males and 58 (38.2%) females, with an average age of 56 (range 28 - 83). There were 108 (71.1%) adenocarcinoma (AD), 30 (19.7%) squamous cell carcinoma (SCC), and 11 (7.2%) small cell lung cancer (SCLC). Most patients (62 cases, 40.8%) had stage IV disease, followed by stage I (39cases, 25.7%) and stage III (37 cases, 24.3%), 14 patients (9.2%) had stage II disease. For 871 sporadic lung cancer patients, average age was 58 (range 27–85). There were 532 (61.1%) AD cases, 236 (27.1%) SCC cases and 84 (9.6%) SCLC cases; majority had stage IV disease (295 cases, 33.9%), followed by stage III (267 cases, 30.7%), and 311 (35.5%) patients were in stage I or II.

**Table 1:** Clinicalpathological characteristics of 1,023 lung cancer patients.

**Variables**	**Total**	**Family history of lung cancer**		***P* value^a^**
		**Positive**	**Negative**
Total number of patients	1,023	152 (14.86%)	871 (85.14%)
Gender				0.29
Male	671 (65.59%)	94 (61.84%)	577 (66.25%)
Female	352 (34.41%)	58 (38.16%)	294 (33.75%)
Average age: 58 years (range 27–85)				0.03
≧50 years	822 (80.35%)	112 (73.68%)	710 (81.52%)
<50 years	201 (19.65%)	40 (26.32%)	161 (18.48%)
Blood type				0.39
A	344 (33.63%)	43 (28.29%)	301 (34.56%)
B	307 (30.01%)	46 (30.26%)	261 (29.97%)
AB	103 (10.07%)	16 (10.53%)	87 (9.99%)
O	269 (26.29%)	47 (30.92%)	222 (25.49%)
Smoking history				0.53
Yes (Current or Ex-smoker)	481 (47.02%)	75 (49.34%)	406 (46.61%)
Never	542 (52.98%)	77 (50.66%)	465 (53.39%)
Anatomy site				0.02^b^
Left lung	463 (45.26%)	82 (53.95%)	381 (43.74%)
Right lung	550 (53.76%)	68 (44.74%)	482 (55.34%)
Bilateral lung	7 (0.69%)	1 (0.66%)	6 (0.69%)
Main bronchus	3 (0.29%)	1 (0.66%)	2 (0.23%)
Histology type				0.13
Adenocarcinoma	640 (62.56%)	108 (71.05%)	532 (61.08%)
Squamous cell carcinoma	266 (26.00%)	30 (19.74%)	236 (27.10%)
Small cell lung cancer	95 (9.29%)	11 (7.24%)	84 (9.64%)
Others	22 (2.15%)	3 (1.97%)	19 (2.18%)
Stage				0.26
I	251 (24.54%)	39 (25.66%)	212 (24.34%)
II	111 (10.84%)	14 (9.21%)	97 (11.14%)
III	304 (29.72%)	37 (24.34%)	267 (30.65%)
IV	357 (34.90%)	62 (40.79%)	295 (33.87%)
Distant organ metastasis				0.12
Present	227 (22.19%)	41 (26.97%)	186 (21.35%)
Absent	796 (77.81%)	111 (73.03%)	685 (78.65%)

a *For categorical variables, using Chi-square test or Fisher's exact test, when there is a cell frequency < 5*.

b *p-value calculated for left/right lung only, other sites are not included*.

### Familial Lung Cancer Subjects of Highland Population: Early Onset; Increased Frequency of: Female, Adenocarcinoma and Stage IV; Unbalance in Anatomic Sites

**Figure 1:**
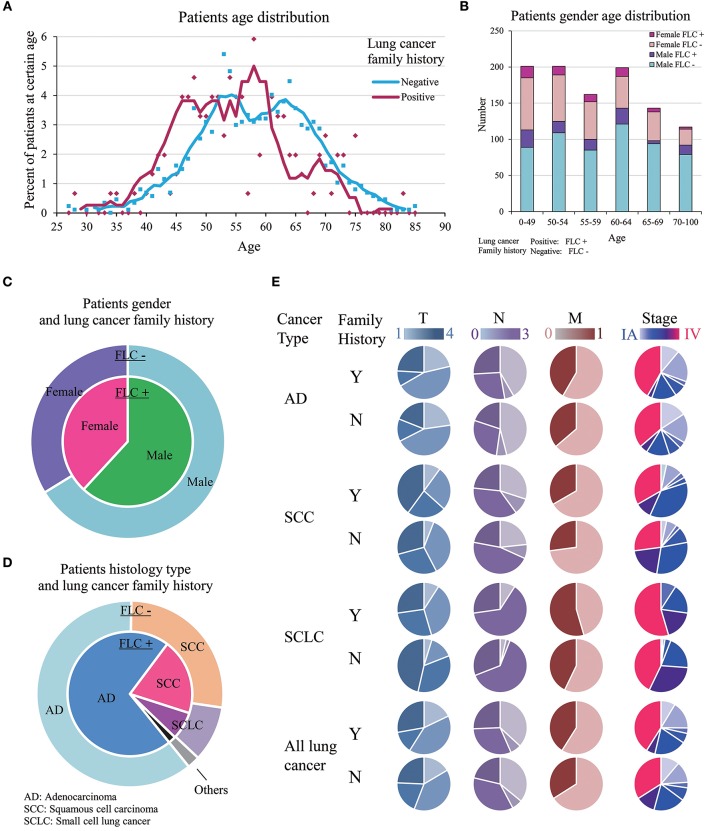
Clinic-pathological features of familial and sporadic lung cancer in highland population. **(A)** Patient age at diagnosis. Both curves fit Mov. Avg. (*E* = 5). There were more individuals under age 50 in FLC group. FLC cases showed two peaks (46 vs. 58 years); sporadic cases also had double peaks (53 vs. 63 years), but both were later than FLC counterpart. (B) Patient gender-age distribution of FLC and sporadic group. **(C)** Patient gender and lung cancer family history. **(D)** Patient histology type and lung cancer family history. **(E)** Patient TNM stage in AD, SCC, and SCLC. Stage IV and M1 were increased in all FLC subgroups, T4 and N3 raised only in AD and SCC. Y, Yes; N, No.

Highland FLC population was compared with the sporadic counterpart, importantly no significant difference was found in smoking status. Considering age at diagnosis, there were more individuals under age 50 in FLC group, and more older patients (≧ 63 years) in sporadic group (Table 1, Figure 1A). Interestingly, FLC group showed two peaks (46 vs. 58 years), sporadic group also had double peaks (53 vs. 63 years), but both were later than FLC counterpart (Figure 1A), it could suggest other potential risk factors genetically or environmentally. Each age section included more than 100 subjects with most sections around 150, reflecting nearly even distribution of patients in each age group; the number of FLC females started to decrease after age 65, while FLC males dropped slower in older age (Figure 1B).

Even not significant, the ratios of: female, AD histology, stage IV and other organ metastasis were all higher in FLC group (Figures 1C–E). Interestingly, significant difference was found on anatomic sites, FLC patients had cancer on left lung more frequent (54.7 vs. 44.2%, *P* = 0.02). In total, ratios of stage T4, N3, M1, and IV all increased in FLC subjects, potentially reflecting higher cancer aggressiveness. Further divided by histologic type (AD, SCC, SCLC), stage IV and M1 were found increased in all subgroups with FLC, while stage T4 and N3 raised only in AD and SCC with FLC (Figure 1E).

**Figure 2:**
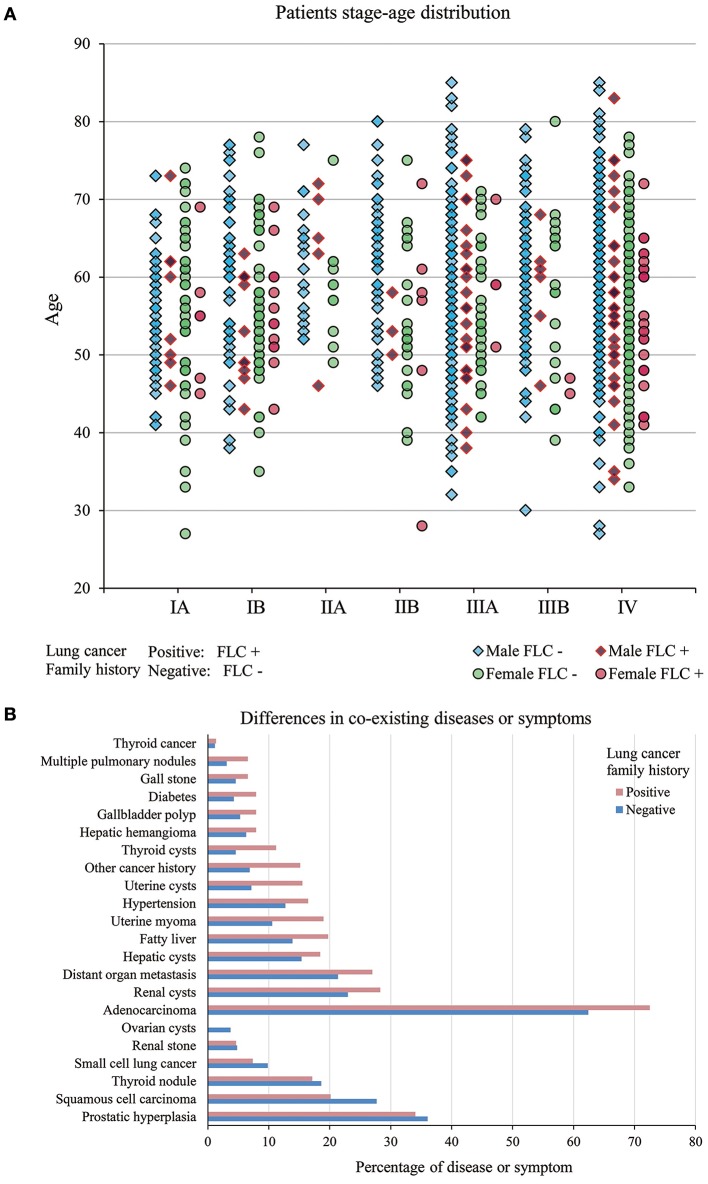
**(A)** Patients stage-age distribution of FLC and sporadic individuals. In FLC group, more males were found in stage IIIA and IV, and females were accumulated in stage IV. For sporadic group, males were enriched in stage IIIA and IV, but stage IA, IB, IIA, IIB all had much more males than their FLC counterpart, while many females were in stage IV, high number of females were also found in stage IA and IB. **(B)** Difference in co-existing diseases or symptoms. In total, 15 had higher frequency in FLC group (including: multiple pulmonary nodules, distant organ metastasis, other cancer history, diabetes, hypertension, thyroid cysts, thyroid cancer, hepatic cysts, fatty liver, hepatic hemangioma, renal cysts, gallbladder polyp, gall stone, uterine myoma, uterine cysts) and only 4 (prostatic hyperplasia, thyroid nodule, renal stone, ovarian cysts) were lower in FLC patients.

Evaluated by gender, age, and stage together, for most stages, the patient age ranged from 30 years to around 80 years, only stage IIA had less subjects. Majority of patients were in stage IIIA to IV. In FLC group, more males were found in stage IIIA and IV, compared with males in other stages, while females were accumulated in stage IV. For sporadic subjects, males were also enriched in stage IIIA and IV, but stage IA, IB, IIA, IIB all had much more males than their FLC counterpart, while many females were in stage IV, high number of females were also found in stage IA and IB (Figure 2A).

### Unbalanced Distribution of Co-existing Diseases Between Familial and Sporadic Lung Cancer in Highland Population

We also investigated other co-existing diseases or symptoms in our subjects (Table S1). In total, 15 had higher frequency in FLC group (including: multiple pulmonary nodules, distant organ metastasis, other cancer history, diabetes, hypertension, thyroid cysts, thyroid cancer, hepatic cysts, fatty liver, hepatic hemangioma, renal cysts, gallbladder polyp, gall stone, uterine myoma, uterine cysts) and only 4 (prostatic hyperplasia, thyroid nodule, renal stone, ovarian cysts) were lower in FLC patients (Figure 2B).

**Figure 3:**
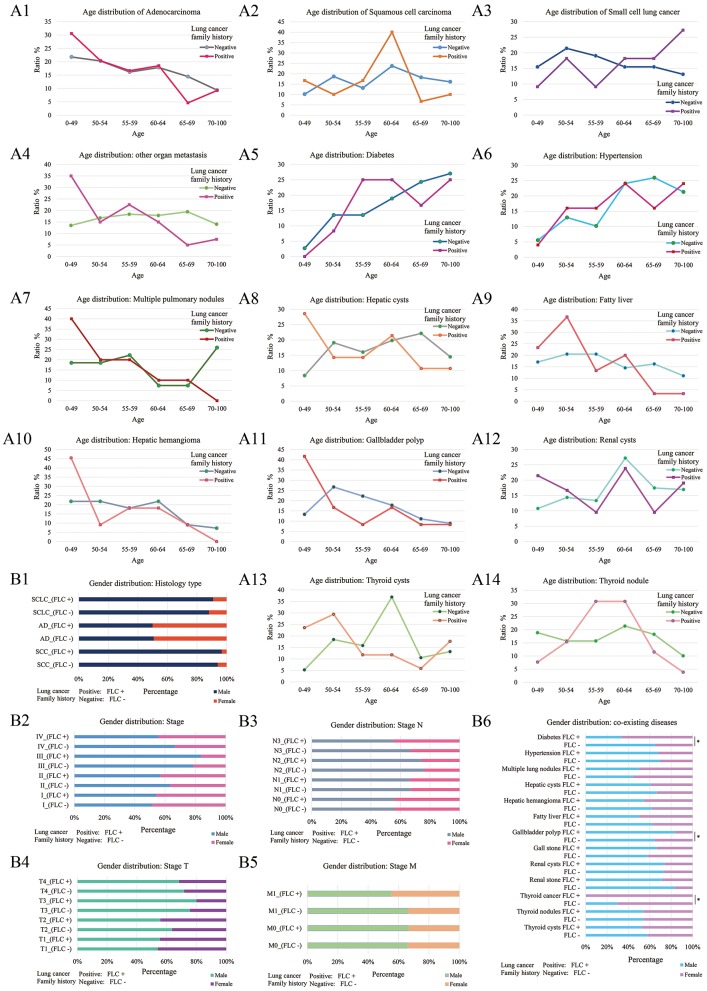
Age and gender distribution of co-existing diseases or symptoms. Divided by age, 7 showed the major increase under age 50 for FLC population (including: AD histology, multiple pulmonary nodules, distant organ metastasis, hepatic cysts, hepatic hemangioma, renal cysts, gallbladder polyp) **(A1,A4,A7,A8,A10–12)**; fatty liver had major increase under age 55 in FLC group **(A9)**; 3 had no apparent age distribution variation between familial and sporadic group (including: SCC histology, diabetes, hypertension) **(A2,A5,A6)**; the rate of FLC patients with SCLC histology seemed to be higher in older age **(A3)**; thyroid cysts also had major increase under age 55 for FLC group, but thyroid nodule had the major peak around age 55–64 **(A13,A14)**. Divided by gender, for histologic type, no apparent gender differences were found between FLC and sporadic group **(B1)**; for TNM stage, there were more females in T2, T4, N3, M1, and stage IV in FLC group **(B2–5)**; when considering co-existing diseases, 6 showed more females with FLC (including: diabetes, thyroid cancer, hepatic cysts, hepatic hemangioma, fatty liver, renal stone); while more FLC males were found with gallbladder polyp, gall stone and multiple pulmonary nodules; others didn't show clear gender bias; only diabetes, thyroid cancer and gallbladder polyp were statistically significant **(B6)**.

Further divided by age, in FLC population, seven showed clear ratio increase in younger patients (age < 50) (including: AD histology, multiple pulmonary nodules, distant organ metastasis, hepatic cysts, hepatic hemangioma, renal cysts, gallbladder polyp) (Figures 3A1,A4,A7,A8,A10–12); while fatty liver and thyroid cysts had major ratio increase under age 55, thyroid nodule had the main ratio peak around age 55-64 (Figures 3A9,A13,A14); the rate of FLC patients with SCLC histology seemed to be higher in older age (Figure 3A3); three had no apparent age distribution variation between familial and sporadic group (including: SCC histology, diabetes, hypertension) (Figures 3A2,A5,A6).

Further divided by gender, for histologic type, no apparent gender difference was found between FLC and sporadic group (Figure 3B1); for TNM stage, even not significant, there were more females in T2, T4, N3, M1, and stage IV in FLC group (Figures 3B2–5); when considering co-existing diseases, six had more FLC females (including: diabetes, thyroid cancer, hepatic cysts, hepatic hemangioma, fatty liver, renal stone); while more FLC males were found with gallbladder polyp, gall stone and multiple pulmonary nodules; only diabetes, thyroid cancer and gallbladder polyp were statistically significant (Figure 3B6).

### Comparison Between Highland Lung Cancer With Other Lung Cancer Population

**Figure 4:**
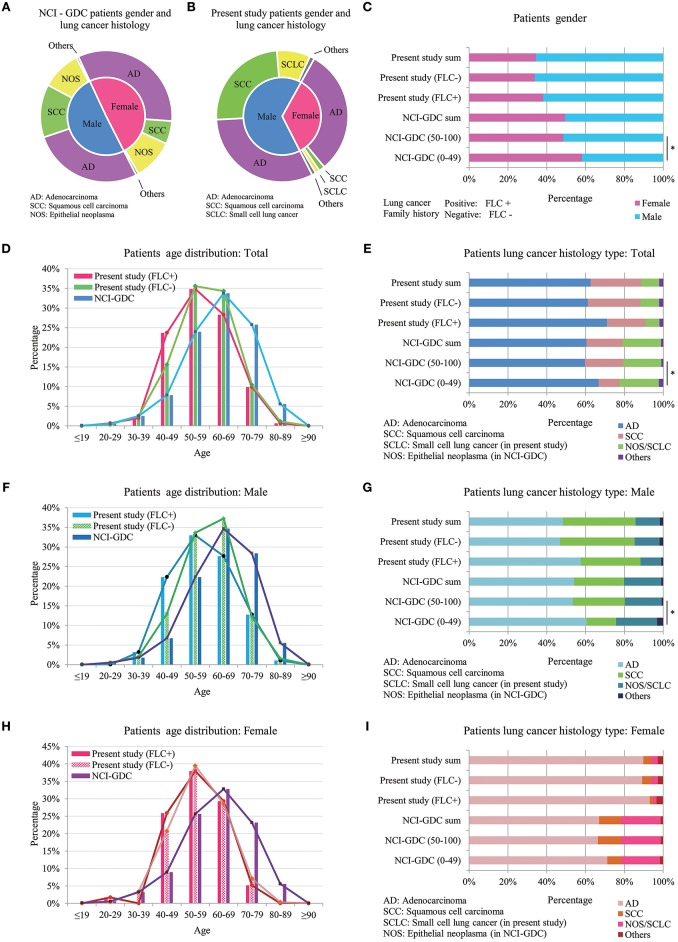
Comparison between highland lung cancer with NCI-GDC lung cancer population. **(A)** Gender and lung cancer histology distribution in GDC patients; **(B)** Gender and lung cancer histology distribution in present study; In GDC population, 0–49 years subjects had statistically higher female ratio, similar to FLC patients of present work **(C)**; Younger GDC patients also showed increased AD histology for male, female and in total **(E,G,I)**. **(D)** In total, GDC subjects developed lung cancer at much older age (peak: 60–69) than both the FLC group (peak: 50–59) and the sporadic group (peak ≦ 60) of present study; **(F)** In males, age distribution of the 3 groups was similar to that of total population, the main peak of our sporadic males moved to higher than 60 years. **(H)** In females, the age curves of our familial and sporadic females almost overlapped with each other, the only difference was at age 40–49.

Present study was compared with data from NCI-GDC (https://portal.gdc.cancer.gov). In total 4754 lung cancer patients (male: 2401, female: 2349, unreported: 4) were included (Table S2). Gender and lung cancer histology had relatively even distribution in GDC patients, but in present study, female patients were clearly dominated by AD histology (Figure 4A,B). Because record of lung cancer family history is not available in GDC data, and according to most references, early-onset is one crucial feature of familial lung cancer (1, 2, 4, 6), furthermore, present study found the major age difference between our familial and sporadic group was around age 50 (Figure 1A), so we divided the GDC lung cancer population into 0–49 and 50–100 years for further comparison.

Compared with present study, divided GDC patients had similar gender and lung cancer histology distribution. In GDC population, 0–49 years subjects had statistically higher female ratio, similar to FLC patients of present work (Figure 4C); moreover, younger GDC patients also showed increased AD histology for male, female and in total (Figures 4E,G,I).

Age was the most interesting. In total, GDC subjects developed lung cancer at much older age (peak: 60–69) than not only the FLC group (peak: 50–59), but also the sporadic group (peak ≦ 60) of present study (Figure 4D), indicating the overall early-onset of our subject population. Importantly, the biggest difference happened in 40–49 age section, the ratio of our FLC patients was 3 times higher than GDC population, while our sporadic group had nearly 2 times higher ratio than GDC subjects. In males, age distribution of the 3 groups was similar to that of total population, and age 40–49 still held the biggest variation, except the main age peak of our sporadic males moved to higher than 60 years (Figure 4F). In females, the age peak was still in 60–69 for GDC population, while the age curve of our familial and sporadic females almost overlapped (peak: 50–59), the only ratio difference was in age 40–49: FLC females was a little higher than the sporadic part, but both were more than 2 times higher in ratio than GDC females (Figure 4H). All together, the results suggest: our subject population may harbor gender-specific or age-specific characters.

A brief comparison on overall survival and mutation spectrum was carried for GDC population (Figure S1). No apparent survival difference was found between 0–49 and 50–100 age groups, but there were small variations in mutation for different age and gender groups. Since present study didn't include survival and mutation data, according to previous reports (19–21), there could be potential mutation differences between familial and sporadic lung cancer in highland population.

## Discussion

Relatives of cancer patients are at an increased risk for the same cancer and also other cancers, among the 27 most common cancers, significant risk ratios were found for pancreatic (2.31), lung (1.69), kidney (1.98), nervous system (1.79), and thyroid cancers (3.28) (2, 22). Two meta-analysis performed on more than 60 studies reported an approximately 2-fold increase associated with family history of lung cancer (23, 24). One investigation conducted by the International Lung Cancer Consortium (ILCCO) in 2012 including 24,000 cases and 23,000 controls, reported a significant 1.5-fold increase in risk due to family history after adjustment for environmental confounders (6); this study also confirmed a higher risk in African-Americans (2.09-fold risk) than Caucasians (1.53-fold risk). Importantly, inherited risks can be further amplified by environmental carcinogen exposure (7).

Younger age at diagnosis is the typical character of familial cancers (25–27). Present study found similar but more complex results (Figure 1A). Although FLC and sporadic group had a small average age gap (56 vs. 58), FLC group included significantly more patients under age 50, and both showed double peaks (FLC: 46 vs. 58; sporadic: 53 vs. 63). This could indicate contribution from multiple factors or existing of subgroups in each population. Firstly, it may reflect different carcinogen exposure at various levels; secondly, since family history is not equal to susceptibility, there can be susceptible individuals in sporadic group and non-susceptible individuals in FLC group; both may cause age peak to split. Noticeably, when compared with GDC data, our subject population showed apparent overall early-onset, it may be explained by higher carcinogen exposure or genetic background variation at population level.

FLC patients tended to have more AD histology, other cancer history, distant organ metastasis and stage IV cases, the latter two are indicator of increased cancer aggressiveness. Studies on young lung cancer patients also supported high AD ratio, prevalence of female and diagnosed in advanced stages (25, 26, 28, 29). Other interesting results included: the age curve of our FLC and sporadic males separated clearly, while age curve of our FLC and sporadic females almost overlapped with each other (Figures 4F,H). One study observed higher risks for female relatives of female proband vs. male relatives of male proband (30); another also supported first-degree female relatives seemed to have a higher risk than first-degree male relatives (4); the third one found female gender and FLC were the most important predictor of lung cancer (8); all reflecting the possibly gender-specific or age-specific characters in different FLC populations.

Regarding to co-existing diseases or symptoms (Table S1, Figure 3), there were also gender-specific or age-specific variations between FLC and sporadic group. FLC patients were more likely to develop benign lesions (polyps, nodules, cysts) early in life. Especially high ratio and early-growth of multiple pulmonary nodules, indicating unhealthy of the lung even before cancer developed. Diabetes and hypertension are typical diseases with family history, and their frequency also increased in our FLC group compared with sporadic counterpart, with diabetes statistically higher in females.

## Conclusion

Present study revealed the clinicopathologic characteristics of familial and sporadic lung cancer in highland population of China, discovered complex differences in gender, age, histologic type, TNM stage and co-existing diseases or symptoms. Future task is to identify hereditary factors which influence lung cancer risk, and also highlight the significance of lung cancer susceptibility screen in our population.

## Author Contributions

YC and XD conception and design. JY, HNiu, and GL data acquisition, data analysis, and interpretation. YC, XD, JY, RH, JZ, and HNin drafting the article and revising it for important intellectual content.

### Conflict of Interest Statement

The authors declare that the research was conducted in the absence of any commercial or financial relationships that could be construed as a potential conflict of interest.

## Supplementary Material

Figure S1 Comparison on overall survival and mutation spectrum in GDC population. **(A)** overall survival (age:0–49); **(B)** overall survival (age: 50–100); **(C)** Mutations: male (age: 0–49); **(D)** Mutations: female (age: 0–49); **(E)** Mutations: male (age: 50–100); **(F)** Mutations: female (age: 50–100).

Table S1 Other co-existing diseases or symptoms in 1,023 lung cancer patients.

Table S2 Clinicopathologic characteristics of 4,754 lung cancer patients from NCI-GDC.

## Abbreviations

FLC: familial lung cancer
AD: Adenocarcinoma
SCC: squamous cell carcinoma
SCLC: small cell lung cancer.
