# Supplementary figures and images for: Characteristics of Familial Lung Cancer in Yunnan-Guizhou Plateau of China
Source: Front Oncol. 2018 Dec 18;8:637. doi: 10.3389/fonc.2018.00637 (PMC6305406; doi:10.3389/fonc.2018.00637)

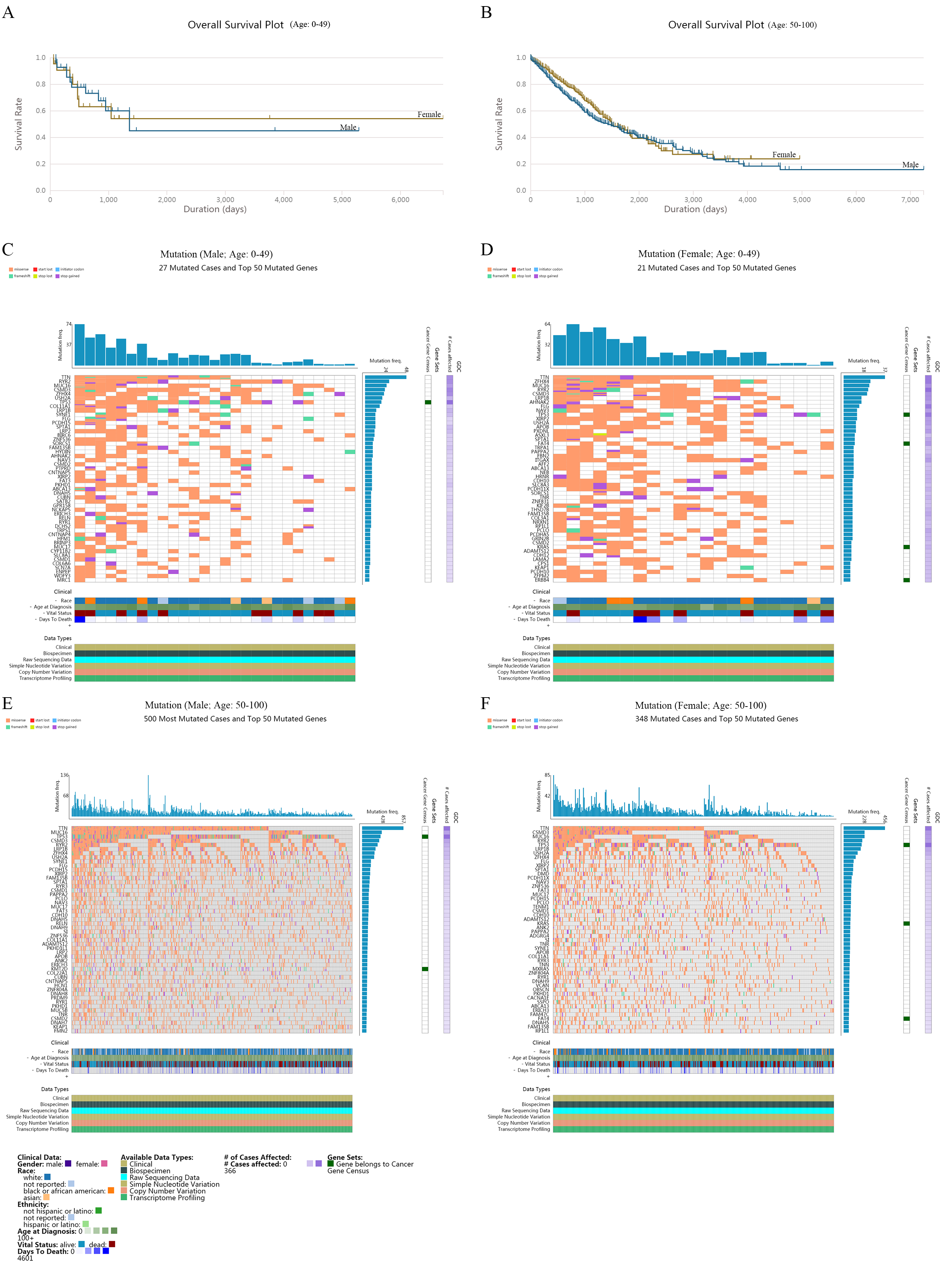

Supplement: Figure S1 — Comparison on overall survival and mutation spectrum in GDC population. (A) overall survival (age:0–49); (B) overall survival (age: 50–100); (C) Mutations: male (age: 0–49); (D) Mutations: female (age: 0–49); (E) Mutations: male (age: 50–100); (F) Mutations: female (age: 50–100). [file Image_1.TIF]
