# Supplementary material for: Characteristics of Familial Lung Cancer in Yunnan-Guizhou Plateau of China
Source: Front Oncol. 2018 Dec 18;8:637. doi: 10.3389/fonc.2018.00637 (PMC6305406; doi:10.3389/fonc.2018.00637)
Supplement: Table S1 — Other co-existing diseases or symptoms in 1,023 lung cancer patients. [file Table_1.docx]

**Table S1. Other co-existing diseases or symptoms in 1023 lung cancer patients**

| No. | Other Diseases or Symptoms | All | Family history of lung cancer | | *P* value ^a^ |
| --- | --- | --- | --- | --- | --- |
|  |  |  | Positive | Negative |  |
|  | Total number of patients | 1023 | 152 (14.86%) | 871 (85.14%) |  |
| 1 | Other cancer history |  |  |  | 0.003 |
|  | Present | 144 (14.08%) | 33 (21.71%) | 111 (12.74%) |  |
|  | Absent | 879 (85.92%) | 119 (78.29%) | 760 (87.26%) |  |
| 2 | Multiple pulmonary nodules |  |  |  | 0.03 |
|  | Present | 37 (3.62%) | 10 (6.58%) | 27 (3.10%) |  |
|  | Absent | 986 (96.38%) | 142 (93.42%) | 844 (96.90%) |  |
| 3 | Diabetes |  |  |  | 0.05 |
|  | Present | 49 (4.79%) | 12 (7.89%) | 37 (4.25%) |  |
|  | Absent | 974 (95.21%) | 140 (92.11%) | 834 (95.75%) |  |
| 4 | Hypertension |  |  |  | 0.22 |
|  | Present | 136 (13.29%) | 25 (16.45%) | 111 (12.74%) |  |
|  | Absent | 887 (86.71%) | 127 (83.55%) | 760 (87.26%) |  |
| 5 | Hepatic cysts |  |  |  | 0.34 |
|  | Present | 162 (15.84%) | 28 (18.42%) | 134 (15.38%) |  |
|  | Absent | 861 (84.16%) | 124 (81.58%) | 737 (84.62%) |  |
| 6 | Hepatic hemangioma |  |  |  | 0.47 |
|  | Present | 67 (6.55%) | 12 (7.89%) | 55 (6.31%) |  |
|  | Absent | 956 (93.45%) | 140 (92.11%) | 816 (93.69%) |  |
| 7 | Fatty liver |  |  |  | 0.06 |
|  | Present | 151 (14.76%) | 30 (19.74%) | 121 (13.89%) |  |
|  | Absent | 872 (85.24%) | 122 (80.26%) | 750 (86.11%) |  |
| 8 | Gall stone |  |  |  | 0.30 |
|  | Present | 50 (4.89%) | 10 (6.58%) | 40 (4.59%) |  |
|  | Absent | 973 (95.11%) | 142 (93.42%) | 831 (95.41%) |  |
| 9 | Gallbladder polyp |  |  |  | 0.20 |
|  | Present | 58 (5.67%) | 12 (7.89%) | 46 (5.28%) |  |
|  | Absent | 965 (94.33%) | 140 (92.11%) | 825 (94.72%) |  |
| 10 | Thyroid cancer |  |  |  | 0.70 |
|  | Present | 12 (1.17%) | 2 (1.32%) | 10 (1.15%) |  |
|  | Absent | 1011 (98.83%) | 150 (98.68%) | 861 (98.85%) |  |
| 11 | Thyroid nodule |  |  |  | 0.66 |
|  | Present | 188 (18.38%) | 26 (17.11%) | 162 (18.60%) |  |
|  | Absent | 835 (81.62%) | 126 (82.89%) | 709 (81.40%) |  |
| 12 | Thyroid cysts |  |  |  | 0.001 |
|  | Present | 57 (5.57%) | 17 (11.18%) | 40 (4.59%) |  |
|  | Absent | 966 (94.43%) | 135 (88.82%) | 831 (95.41%) |  |
| 13 | Renal cysts |  |  |  | 0.15 |
|  | Present | 243 (23.75%) | 43 (28.29%) | 200 (22.96%) |  |
|  | Absent | 780 (76.25%) | 109 (71.71%) | 671 (77.04%) |  |
| 14 | Renal stone |  |  |  | 0.91 |
|  | Present | 49 (4.79%) | 7 (4.61%) | 42 (4.82%) |  |
|  | Absent | 974 (95.21%) | 145 (95.39%) | 829 (95.18%) |  |
| 15 | Ovarian cysts ^b^ |  |  |  | 0.22 |
|  | Present | 11 (3.13%) | 0 (0.00%) | 11 (3.74%) |  |
|  | Absent | 341 (96.87%) | 58 (100.00%) | 283 (96.26%) |  |
| 16 | Uterine myoma ^b^ |  |  |  | 0.07 |
|  | Present | 42 (11.93%) | 11 (18.97%) | 31 (10.54%) |  |
|  | Absent | 310 (88.07%) | 47 (81.03%) | 263 (89.46%) |  |
| 17 | Uterine cysts ^b^ |  |  |  | 0.07 |
|  | Present | 30 (8.52%) | 9 (15.52%) | 21 (7.14%) |  |
|  | Absent | 322 (91.48%) | 49 (84.48%) | 273 (92.86%) |  |
| 18 | Prostatic hyperplasia ^c^ |  |  |  | 0.71 |
|  | Present | 240 (35.77%) | 32 (34.04%) | 208 (36.05%) |  |
|  | Absent | 431 (64.23%) | 62 (65.94%) | 369 (63.95%) |  |

^a^ For categorical variables, using Chi-square test or Fisher’s exact test, when there is a cell frequency less than 5.

^b^ Ovarian cysts, uterine cysts and uterine myoma calculated only in females.

^c^ Prostatic hyperplasia calculated only in males.
