# Supplementary material for: Characteristics of Familial Lung Cancer in Yunnan-Guizhou Plateau of China
Source: Front Oncol. 2018 Dec 18;8:637. doi: 10.3389/fonc.2018.00637 (PMC6305406; doi:10.3389/fonc.2018.00637)
Supplement: Table S2 — Clinicopathologic characteristics of 4,754 lung cancer patients from NCI-GDC. [file Table_2.docx]

**Table S2. Clinicalpathological characteristics of 4754 lung cancer patients from NCI-GDC^a^**

| **Variables** | **Total** | **Patients age** | | ***P* value ^b^** |
| --- | --- | --- | --- | --- |
|  |  | **0 - 49 years** | **50 - 100 years** |  |
| Total number of patients | 4754 | 520 | 4234 |  |
| Gender |  |  |  | 0.005 |
| Male | 2401 | 218 | 2183 |  |
| Female | 2349 | 302 | 2047 |  |
| unreported | 4 | 0 | 4 |  |
| Age: Total |  |  |  |  |
| ≤19 | 3 | 3 | -- |  |
| 20-29 | 26 | 26 | -- |  |
| 30-39 | 119 | 119 | -- |  |
| 40-49 | 372 | 372 | -- |  |
| 50-59 | 1139 | -- | 1139 |  |
| 60-69 | 1605 | -- | 1605 |  |
| 70-79 | 1225 | -- | 1225 |  |
| 80-89 | 264 | -- | 264 |  |
| ≥90 | 1 | -- | 1 |  |
| Age: Male |  |  |  |  |
| ≤19 | 0 | 0 | -- |  |
| 20-29 | 13 | 13 | -- |  |
| 30-39 | 43 | 43 | -- |  |
| 40-49 | 162 | 162 | -- |  |
| 50-59 | 536 | -- | 536 |  |
| 60-69 | 832 | -- | 832 |  |
| 70-79 | 681 | -- | 681 |  |
| 80-89 | 133 | -- | 133 |  |
| ≥90 | 1 | -- | 1 |  |
| Age: Female |  |  |  |  |
| ≤19 | 3 | 3 | -- |  |
| 20-29 | 13 | 13 | -- |  |
| 30-39 | 76 | 76 | -- |  |
| 40-49 | 210 | 210 | -- |  |
| 50-59 | 602 | -- | 602 |  |
| 60-69 | 770 | -- | 770 |  |
| 70-79 | 544 | -- | 544 |  |
| 80-89 | 131 | -- | 131 |  |
| ≥90 | 0 | -- | 0 |  |
| Histology type: Total |  |  |  | 0.000 |
| Adenocarcinoma | 2874 | 347 | 2527 |  |
| Squamous cell carcinoma | 886 | 55 | 831 |  |
| Epithelial neoplasma | 938 | 106 | 832 |  |
| Others | 56 | 12 | 44 |  |
| Histology type: Male |  |  |  | 0.000 |
| Adenocarcinoma | 1298 | 132 | 1166 |  |
| Squamous cell carcinoma | 618 | 33 | 585 |  |
| Epithelial neoplasma | 459 | 46 | 413 |  |
| Others | 26 | 7 | 19 |  |
| Histology type: Female |  |  |  | 0.089 |
| Adenocarcinoma | 1573 | 215 | 1358 |  |
| Squamous cell carcinoma | 268 | 22 | 246 |  |
| Epithelial neoplasma | 478 | 60 | 418 |  |
| Others | 30 | 5 | 25 |  |

^a^ Infromation obtained from NCI Genomic Data Commons (GDC) Data Portal (https://portal.gdc.cancer.gov)

^b^ For categorical variables, using Chi-square test or Fisher’s exact test, when there is a cell frequency less than 5.
